# Comparisons of Different Screening Tools for Identifying Fracture/Osteoporosis Risk Among Community-Dwelling Older People

**DOI:** 10.1097/MD.0000000000003415

**Published:** 2016-05-20

**Authors:** Sy-Jou Chen, Yi-Ju Chen, Chui-Hsuan Cheng, Hei-Fen Hwang, Chih-Yi Chen, Mau-Roung Lin

**Affiliations:** From the Department of Emergency Medicine (S-JC), Tri-Service General Hospital, National Defense Medical Center; Graduate Institute of Injury Prevention and Control (S-JC, C-YC, M-RL), College of Public Health and Nutrition, Taipei Medical University; Department of Nursing (Y-JC), Cathay General Hospital, Taipei; Department of Emergency Medicine (C-HC), Taichung Branch, Tzu-Chi General Hospital, Taichung, and Department of Nursing (Hei-FH), National Taipei University of Nursing and Health Science, Taipei, Taiwan.

## Abstract

A prospective study was conducted to compare criterion, predictive, and construct validities of 9 fracture/osteoporosis assessment tools, including calcaneal quantitative ultrasonography (QUS), Age Bulk One or Never Estrogens (ABONE), body weight criterion (BWC), Fracture Risk Assessment Tool (FRAX), Garvan fracture risk calculator (GARVAN), Osteoporosis Risk Assessment Instrument (ORAI), Osteoporosis Index of Risk (OSIRIS), Osteoporosis Self-Assessment Tool for Asians (OSTA), and Simple Calculated Osteoporosis Risk Estimation (SCORE), among older men and women in Taiwan.

Using the femoral neck dual-energy x-ray absorptiometry (DXA) T-score as an external criterion, the sensitivity, specificity, positive and negative predictive values, positive and negative likelihood ratios, and the area under the receiver operating characteristic curve (AUC) for each tool were calculated. The ability of these tools to predict injurious falls was examined. A principal component analysis was applied to understand whether these tools were measuring the same underlying construct.

The FRAX, BWC, ORAI, OSIRIS, OSTA, and SCORE had AUCs of ≥0.8 in men, while the GARVAN, OSIRIS, OSTA, and SCORE had AUCs of ≥0.8 in women. The sensitivity, negative predictive value, and likelihood ratio of the ABONE, BWC, ORAI, OSIRIS, OSTA, and SCORE tools in both men and women were 100%, ≥90%, and 0.0, respectively; the specificity and positive predictive value and likelihood ratio were far from satisfactory. The GARVAN displayed the best predictive ability of a fall in both men (AUCs, 0.653–0.686) and women (AUCs, 0.560–0.567), despite being smaller in women. The 9 screening tools and 2 central DXA measurements assessed 5 different factors, while the ABONE, BWC, ORAI, OSIRIS, OSTA, and SCORE measured the same one.

Simple self-assessment tools can serve as initial screening instruments to rule out persons who have osteoporosis; however, these tools may measure a different construct other than fracture/osteoporosis risk.

## INTRODUCTION

Identifying older adults at high risk for osteoporotic fractures and early interventions can reduce hospital admissions, disabilities, mortality, and economic burdens to society.^1^ Dual-energy x-ray absorptiometry (DXA) scans of central skeletal sites such as the hip and spine are the standard assessment method to diagnose low bone mineral density (BMD).^2^ Nevertheless, central DXA is limited by its high cost, lack of portability, and exposure to ionizing radiation for screening fracture/osteoporosis risk in community-dwelling older people, particularly those living in suburban and rural areas.

Alternatively, distal DXA, biochemical markers of bone resorption and formation, and quantitative ultrasonography (QUS)^3^ were devised in an attempt to provide cost-effective and high-availability methods. Distal DXA poorly predicts a fracture, and bone markers vary widely among individuals. QUS of the calcaneus reflects both bone density and bone quality, and it is portable, radiation-free, and relatively low-cost.^4^ Furthermore, according to clinical risk factors, some fracture/osteoporosis risk self-assessment tools, such as the Age Bulk One or Never Estrogens (ABONE),^5^ body weight criterion (BWC),^6^ Fracture Risk Assessment Tool (FRAX),^7^ Garvan fracture risk calculator (GARVAN),^8^ Osteoporosis Risk Assessment Instrument (ORAI),^9^ Osteoporosis Index of Risk (OSIRIS),^10^ Osteoporosis Self-Assessment Tool for Asians (OSTA),^11^ and Simple Calculated Osteoporosis Risk Estimation (SCORE),^12^ may be suitable for mass screening among community-dwelling older people. However, most of these tools have not been validated in older men, and whether they measure the same construct as central DXA has not been examined.

In this study, clinimetric properties, such as the criterion, predictive, and construct validities of calcaneal QUS, ABONE, BWC, FRAX, GARVAN, ORAI, OSIRIS, OSTA, and SCORE among community-dwelling older men and women in Taiwan were investigated.

## METHODS

### Study Participants

During a 6-month period from July to December 2012, we recruited study participants at community centers in Tanzi District of Taichung City, west-central Taiwan. Persons aged ≥60 years who had a registered household in Tanzi District and who were able to ambulate independently were eligible for the study. Exclusion criteria were difficulty with verbal communications, severe physical restrictions (e.g., severe spondylosis of the spine, joint arthritis of the lower limbs, etc.), and severe cardiopulmonary diseases (e.g., ischemic chest pain, shortness of breath, exertional dyspnea, recurrent dizziness, orthopnea, palpitation, and tachycardia).

In total, 553 community-dwelling older people, consisting of 186 men and 367 women, participated in the baseline assessment conducted at the Taichung branch of Tzu-Chi General Hospital. This research was approved by the Institutional Review Board of Taipei Medical University and Tzu-Chi General Hospital, and written consent was obtained from each participant.

### Data Collection

The baseline assessment included physical measurements and personal interviews. Physical measurements consisted of height, weight, visual acuity, DXA, and QUS. According to the body mass index (BMI) as the weight (kg) divided by the square of the height (m), subjects were categorized as being underweight (<18.5 kg/m^2^), an ideal weight (18.5–23.9 kg/m^2^), overweight (24–26.9 kg/m^2^), or obese (≥27 kg/m^2^).

The areal BMD at the left femoral neck and lumbar spine (anterior-posterior L1–L4), recorded as g/cm^2^ and a T-score,^13^ was measured using a Hologic Discovery Wi Bone Densitometer (Hologic, Bedford, MA). The World Health Organization (WHO) quantitatively defines osteoporosis as a BMD of 2.5 standard deviation units or more below the mean value for young adults (T-score ≤ 2.5).^2^ In participants who had undergone a left hip replacement surgery, the right femoral neck was measured instead. The hydroxyapatite hip phantom and spine phantom with 4 semianthropomorphic hydroxyapatite vertebrae provided by the manufacturer were scanned weekly.

QUS of the left heel was measured using a Lunar Achilles Insight device (GE Lunar, Madison, WI). Three types of parameters are assessed by the device: the speed of sound (SOS), broadband ultrasound attenuation (BUA), and a stiffness index. Sound waves pass faster through higher bone densities and better elasticity, and broadband signals are attenuated by higher bone densities and better bone structures. The stiffness index is a composite parameter, determined by the machine according to the formula of (0.67 × BUA + 0.28 × SOS) − 420.^14^ The heel-estimated BMD (or QUS T-score) was also calculated based on the same young adults as a reference population. The precision of calcaneal QUS was assessed by obtaining 3 measurements after repositioning 20 subjects. The coefficients of variation of SOS, BUA, and heel-estimated BMD were 0.24%, 3.15%, and 3.24%, respectively. Acoustic phantoms provided by the manufacturer were scanned weekly.

Personal interviews collected information on age, gender, education level, marital status, cigarette smoking, alcohol consumption, regular exercise, chronic conditions, medication use, cognitive status, a history of falls and fractures, and other information for calculating risk scores for the 9 fracture/osteoporosis self-assessment tools. The cognitive status was measured using the Mini Mental State Examination with a score of ≤23 indicating impairment.^15^

### Self-Assessment Tools

Eight self-assessment screening tools, including the ABONE, BWC, FRAX, GARVAN, ORAI, OSIRIS, OSTA, and SCORE, calculate a risk score of fracture/osteoporosis for each individual. Self-reported variables were collected using personal interviews with structured questionnaires. Interview procedures and interviewer attitudes were standardized through participation in a 4-hour training course. Risk factors, including calculating the risk score, scoring algorithm, and recommended cutoff points of fracture/osteoporosis for these self-assessment tools, in addition to the QUS, are described in detail in Table 1.

**TABLE 1:**
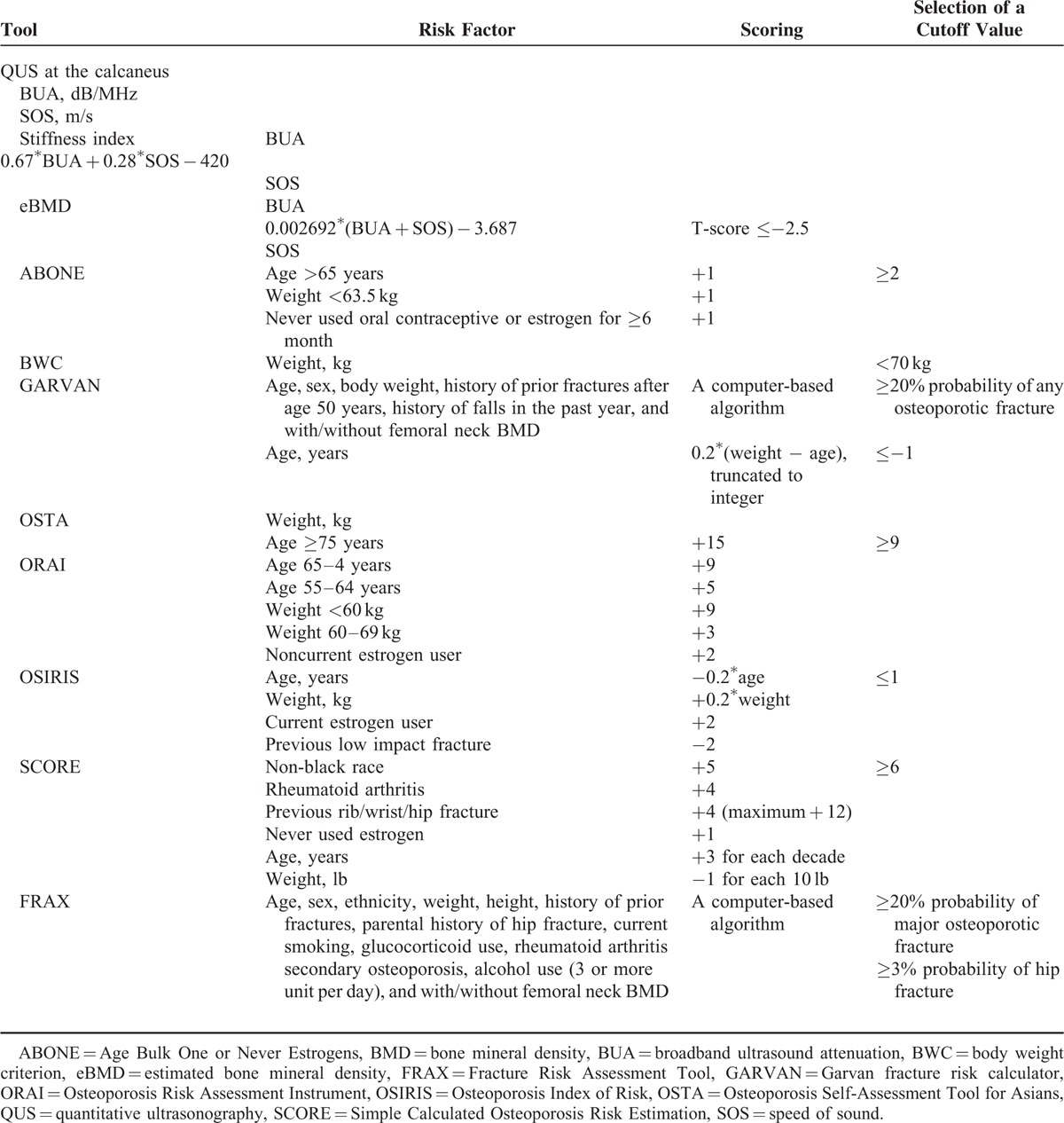
Brief Descriptions of 9 Fracture/Osteoporosis Screening Assessment Tools

The FRAX and GARVAN can be used with or without BMD to calculate the 5- and/or 10-year probability of a hip fracture and other osteoporotic or fragility fractures. For comparison with FRAX, only the 10-year probabilities of a hip fracture and any osteoporotic fracture for GARVAN were computed. Femoral neck DXA BMD data were not included in the calculation of the risk scores of the 2 tools, since this study emphasized screening, and femoral neck DXA BMD was treated as a standard for defining osteoporosis.

### Criterion Validity

The criterion validity detects how closely a QUS parameter or self-assessment tool is related to the femoral neck DXA BMD. Here, the femoral neck DXA T-score was used as an external criterion. The femoral neck DXA BMD is considered the best predictor of hip fracture and is comparable to other measurements for predicting fractures at other sites,^16,17^ and it could discriminate older Taiwanese people with lower-extremity fractures from those without; the areas under the receiver operating characteristic curve (AUC) for the discrimination were 0.821 in men and 0.734 in women.^18^

The sensitivity, specificity, positive and negative predictive values, positive and negative likelihood ratios, and the AUC for each of the QUS parameters and self-assessment tools to discriminate individuals who had osteoporosis from those who did not were calculated. The positive predictive value was the proportion of persons with osteoporosis who had a positive result. The negative predictive value was the proportion of persons without osteoporosis who had a negative result. The positive likelihood ratio (sensitivity/(1 − specificity)) indicates how much more likely a positive screening tool result was obtained for low bone density (i.e., femoral neck DXA-determined osteoporosis) than for normal or subnormal bone density. The negative likelihood ratio ((1 − sensitivity)/specificity) indicates how much less likely a negative screening tool result was obtained for low bone density than for normal bone density. The AUC, which ranges from 0.5 for a noninformative instrument to 1.0 for perfect concurrence, represented a summary measure of the criterion validity.^19^

### Predictive Validity

The predictive validity determines how well a QUS parameter or self-assessment tool predicted the occurrence of an injurious fall during a 12-month follow-up period. The occurrence of a fracture is a rare event; more than 90% of fractures result from falls; ^20^ and predictors for fractures are similar to those for falls.^21^ Accordingly, we used injurious falls as a surrogate for fractures to examine the predictive ability of the fracture/osteoporosis screening tools.

The occurrence of falls was prospectively monitored and recorded daily using a diary mailed monthly to the study coordinator over the 12-month follow-up period. Participants who failed to return the diary or provided incomplete data were contacted by telephone. An injurious fall was defined as an unintentional loss of balance with the body hitting the floor or ground from a standing height or less that resulted in any outpatient or emergency room visit or hospital admission.^22^ The AUC was computed for each screening tool, with a larger AUC indicating a better predictive ability.

### Construct Validity

The assessment tools that conceptually converge should be relatively strongly correlated, whereas those tools with less in common should show weaker correlations. We hypothesized moderate or higher correlations (*r* ≥ 0.4) among the 10 assessment tools. A Pearson correlation coefficient of <0.4 is considered low, one of ≥0.4 and <0.75 moderate, and one of ≥0.75 high.^23^

A principal component analysis with the orthomax rotation method was applied to further understand whether these screening tools and 2 DXA measures of the femoral neck and lumber spine were measuring the same underlying construct (i.e., fracture/osteoporosis risk). The 2 DXA measures were included to check if these screening tools measured the same construct (BMD). Three criteria of factor eigenvalues (>1), the proportion of total variance (>5%), and a scree test indicate how many common factors should be adequate to represent these fracture/osteoporosis assessment tools.^24^ Measurements of these tools were expected to converge into a single common factor if they measured the same underlying construct. Here, loadings of >0.5 of screening tools onto an extracted factor indicated that those tools converged on the underlying construct.^25^

All data analyses were stratified by gender, and all statistical calculations were conducted using the SAS system (SAS Institute, Cary, NC) for Windows vers. 9.3.

## RESULTS

Distributions of demographic and medical characteristics of the 553 participants, consisting of 186 men and 367 women, are shown in Table 2. Of these participants, 66.4% were females; mean ages were 68.1 years in men and 67.1 years in women; 21.7% had attained an educational level of college or above; 75.6% had a spouse present; 54.9% had an intermediate BMI and 42.4% had an abnormal or high BMI; 1.5% were current smokers; 75.2% exercised regularly; 22.1% had impaired visual acuity; 3.3% were cognitively impaired; 53.0% had ≥4 chronic conditions; 30.5% used ≥ 3 medications; and 17.5% had a femoral neck DXA BMD T-score of ≤−2.5. Most distributions of these characteristics differed between men and women.

**TABLE 2:**
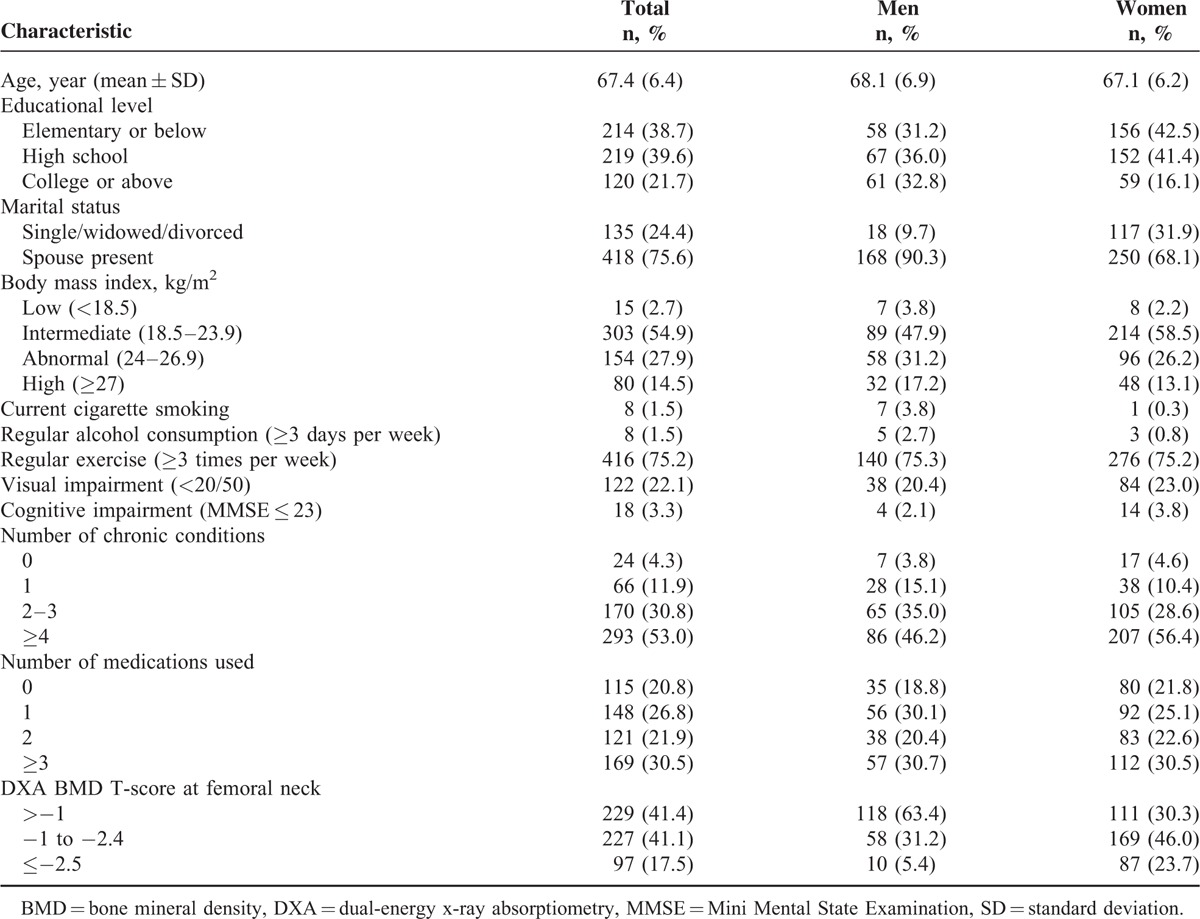
Sociodemographics and Medical Characteristics of the 553 Older Persons, Consisting of 186 Older Men and 367 Older Women

### Criterion Validity

Osteoporosis was defined by a femoral neck DXA BMD T-score of ≤−2.5, and values of the sensitivity, specificity, positive and negative predictive values, positive and negative likelihood ratios, and AUC of the 9 fracture/osteoporosis screening tools are shown in Table 3. In men, the AUCs of QUS parameters varied 0.70 to 0.73, those of FRAX for hip fracture and major osteoporotic fracture varied 0.77 to 0.85, and those of GARVAN varied 0.72 to 0.72. Of the remaining ABONE, BWC, ORAI, OSIRIS, OSTA, and SCORE tools, the sensitivity, negative predictive value, likelihood ratio, and AUC were 100%, 94% to 97%, 0.0, and 0.78 to 0.94, respectively; the specificity, positive predictive value, and positive likelihood ratio were far from satisfactory.

**TABLE 3:**
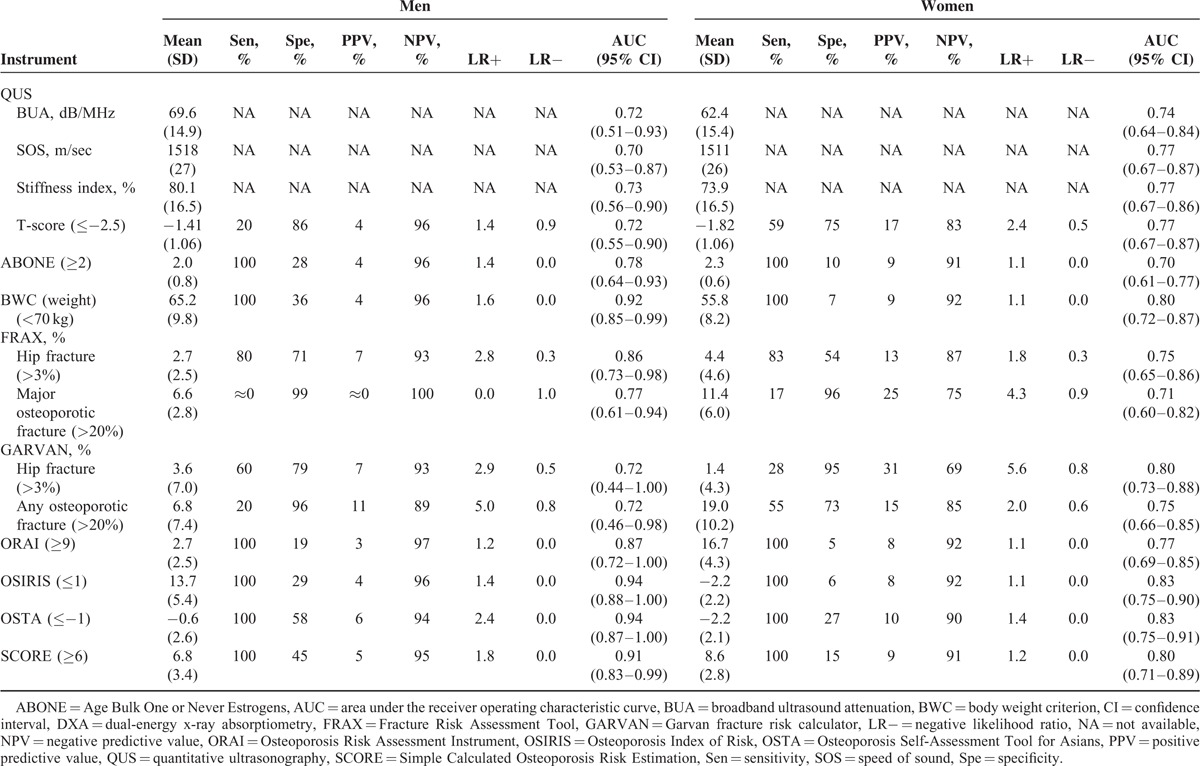
Criterion Validity: Sen, Spe, PPV, NPV, and LR− of QUS, ABONE, BWC, FRAX, GARVAN, ORAI, OSIRIS, OSTA, and SCORE for Femoral Neck DXA-Determined Osteoporosis

In women, the AUCs of QUS parameters ranged 0.74 to 0.77; those of the FRAX ranged 0.71 to 0.75; and those of the GARVAN for hip fracture and osteoporotic fracture ranged 0.75 to 0.80. Of the remaining tools, the sensitivity, negative predictive value, likelihood ratio, and AUC were 100%, 90% to 92%, 0.0, and 0.70 to 0.83, respectively. As a whole, these tools performed better in men than women.

### Predictive Validity

During the 12-month follow-up, 15 men and 48 women experienced an injurious fall. The AUCs of the 9 screening tools used to predict the occurrence of a fall are shown in Table 4. In men, the GARVAN for hip fracture and osteoporotic fracture had the greatest AUCs (0.686 and 0.653), followed by the FRAX for hip fracture and major osteoporotic fracture (0.661 and 0.629), SCORE (0.618), and OSIRIS (0.614) and OSTA (0.614).

**TABLE 4:**
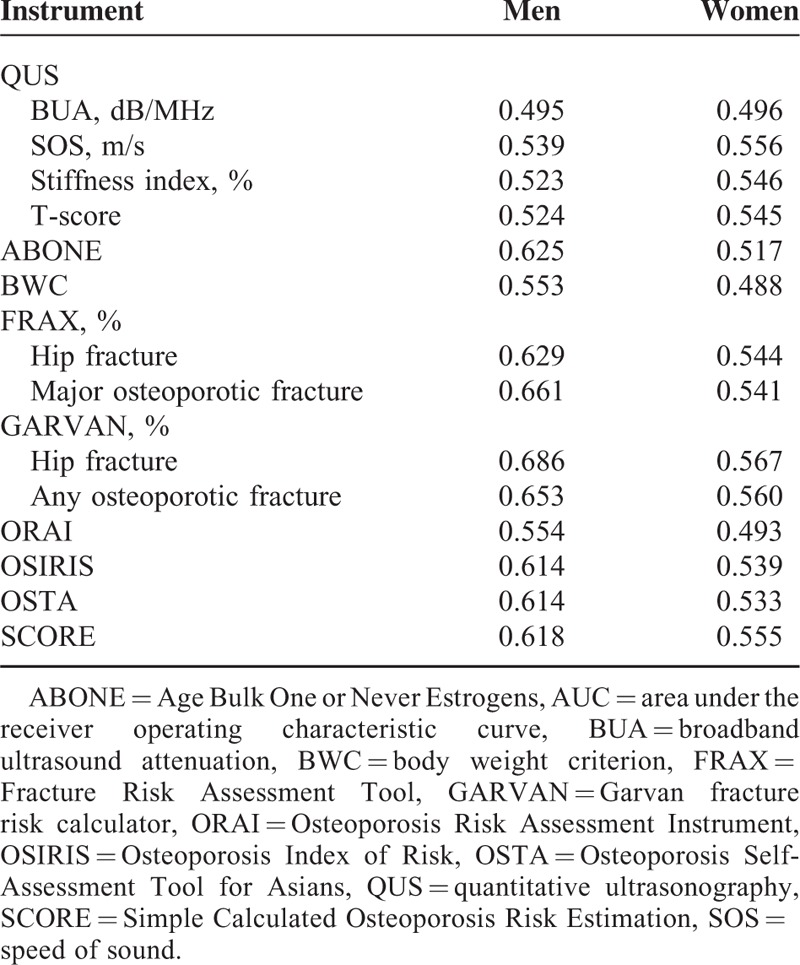
Predictive Validity: AUC for QUS, ABONE, BWC, FRAX, GARVAN, ORAI, OSIRIS, OSTA, and SCORE, According to the Occurrences of an Injurious Fall

In women, the GARVAN for hip fracture and osteoporotic fracture had the greatest AUCs (0.567 and 0.560), followed by the QUS SOS (0.556) and SCORE (0.555).

### Construct Validity

Results of Pearson correlation analysis among the 9 screening tools and 2 DXA measures are shown in Table 5. Moderate or higher correlations were found between the femoral neck DXA BMD and BWC (0.52 in men and 0.47 in women), ORAI (0.43 in men and 0.47 in women), OSIRIS (0.50 in men and 0.54 in women), OSTA (0.50 in men and 0.54 in women), and SCORE (0.45 in men and 0.51 in women), between QUS parameters (0.82–1.00 in men and 0.85–1.00 in women), between 2 FRAX measures and 7 other tools (0.41–0.73 in men and 0.43–0.69 in women), between 2 GARVAN measures and 7 other tools (0.47–0.65 in men and 0.40–0.66 in women), and for the ABONE, BWC, ORAI, OSIRIS, OSTA, and SCORE (0.62–0.99 in men and 0.43–0.98 in women).

**TABLE 5:**
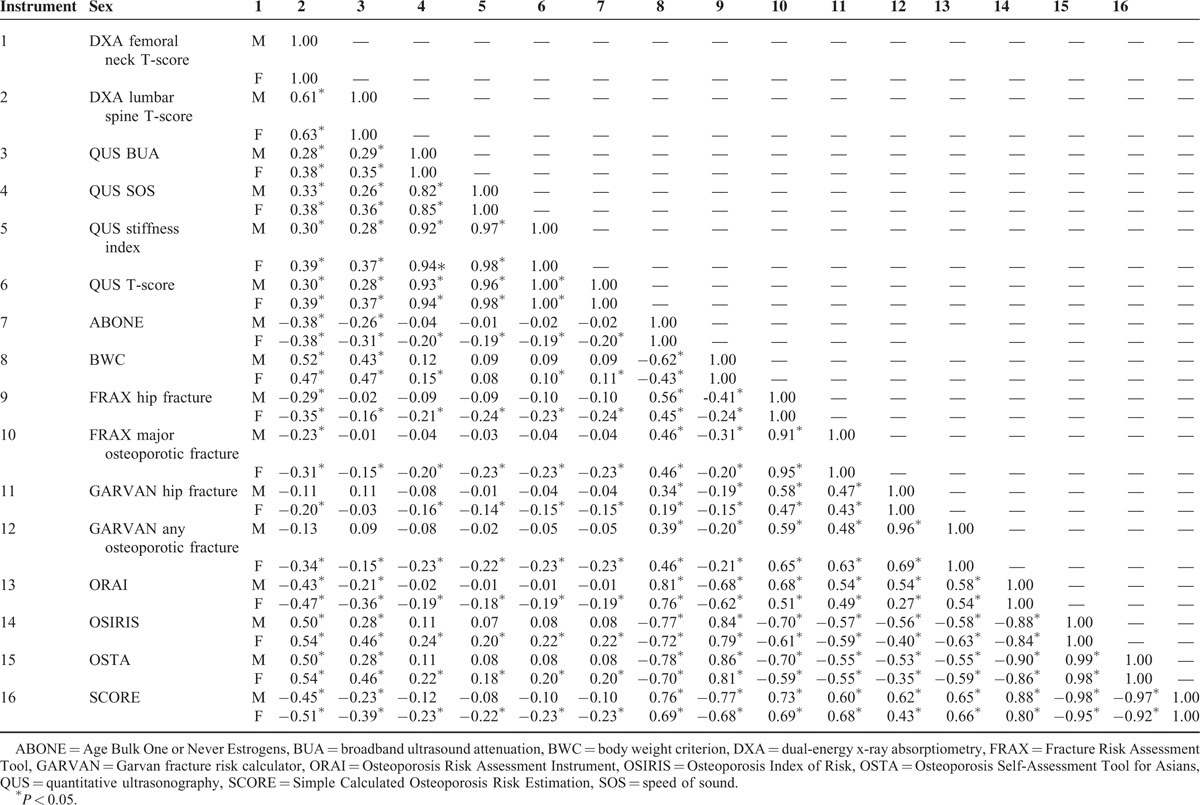
Pearson Correlation Coefficients Among 9 Screening Tools and 2 DXA Measures

Results of the principle components analysis for the construct validity among 9 screening tools and 2 DXA measures are shown in Table 6. According to factor eigenvalues, the proportion of the total variance, and scree test, 5 factors underlying the 9 tools and 2 DXA measures in both men and women were extracted. Specifically, underlying constructs with high loading scores converged on the same factors among the ABONE, BWC, ORAI, OSIRIS, OSTA, and SCORE, between the QUS parameters, between the 2 GARVAN measures, between the 2 FRAX measures, and between femoral neck and lumbar spine DXA T-scores.

**TABLE 6:**
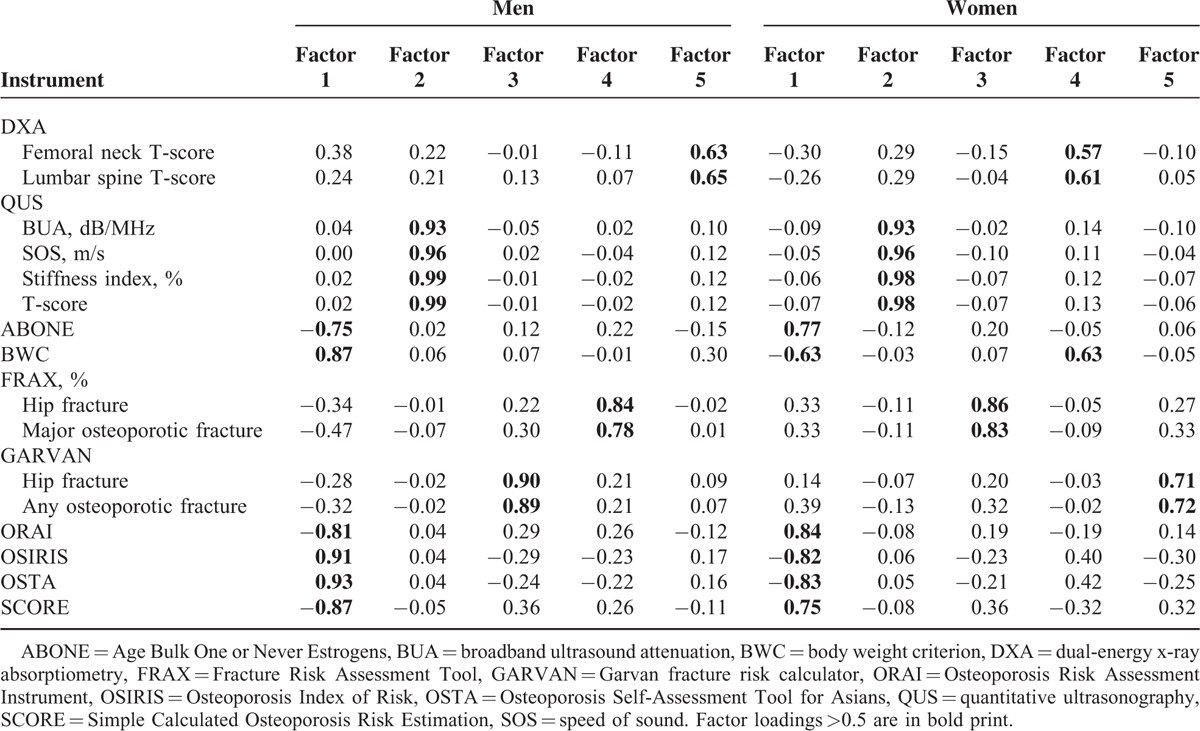
Construct Validity: the Principal Components Analysis for DXA, QUS, ABONE, BWC, FRAX, GARVAN, ORAI, OSIRIS, OSTA, and SCORE

## DISCUSSION

In summary, among the 9 fracture/osteoporosis screening tools, the FRAX, BWC, ORAI, OSIRIS, OSTA, and SCORE had AUC values of ≥0.8 in men, while the GARVAN, OSIRIS, OSTA, and SCORE had AUC values of ≥0.8 in women. The GARVAN displayed the greatest ability to predict a fall in both men and women, despite being smaller in women. The 9 screening tools and 2 central DXA measurements assessed 5 different constructs, and the ABONE, BWC, ORAI, OSIRIS, OSTA, and SCORE measured the same one.

Several features of 6 simple self-assessment tools, including the ABONE, BWC, ORAI, OSIRIS, OSTA, and SCORE, were noted. First, the overall clinimetric performance of these simple assessment tools was not less adequate than the FRAX, GARVAN, and QUS parameters. A recent study even reported that simple tools had better performances than the FRAX with BMD for identifying women with an increased risk of a fracture.^26^ Second, on the basis of the high negative predictive value and likelihood ratio for the criterion validity and low prevalence of fractures, these simple tools are useful in identifying those individuals who do not need BMD testing, particularly among older men. Better results for men of the negative predictive value and likelihood ratio likely reflected their lower prevalence of osteoporosis. Third, unlike the FRAX and GARVAN that directly assess the absolute risk of fracture, all these tools were originally developed to identify osteoporosis risk, and the definition of low BMD may differ among the tools, with the ABONE, BWC, OSTA, and OSIRIS using a T-score of ≤−2.5 and the ORAI and SCORE using a T-score of ≤−2.0. However, this study defined T-scores of ≤−2.5 as osteoporosis for the ORAI and SCORE. Finally, of these 6 self-assessment tools, the OSTA was developed and validated in postmenopausal women in Asian countries, while the others were developed using Caucasian women. The OSTA was also the only tool that has been validated in men.^11,27^ Despite the overall clinimetric performance of some tools such as the OSIRIS being no less adequate than that of the OSTA, the OSTA displayed a higher AUC and specificity than the others, implying that the screening cutoff points in these tools may need to be validated in Asian populations.

The FRAX and GARVAN, compared to osteoporosis screening tools (e.g., the ORAI, OSIRIS, OSTA, and SCORE), had poorer criterion validities for DXA-determined osteoporosis but displayed better predictive ability of a fall. Despite a fracture most likely occurring in the osteoporotic range, the better prediction validities of the FRAX and GARVAN were partly accounted for by certain clinical risk factors (e.g., alcohol intake, rheumatoid arthritis, prior fractures, and falls history) that are also risk factors for falls. This finding also revealed complicated interrelations among osteoporosis, fracture risk, and falls. Owing to a low correlation between osteoporosis and fracture occurrence and a high correlation between falls and fracture occurrence, several investigators advocated that fracture reduction in older people should shift from osteoporosis intervention to fall prevention.^28,29^ Nevertheless, a low bone density may also increase the risk of falling, particularly in older women.^30^ Furthermore, weak correlations were found for the FRAX and GARVAN with other fracture/osteoporosis assessment tools, possibly reflecting that the FRAX and GARVAN were developed to predict fracture risk, while other tools were developed to assess osteoporosis risk.

Calcaneal QUS did not have a better performance for the criterion and prediction validities than did the other screening tools. The nature, precision, reference ranges, and bone site may have confounded the results of the criterion validity. Calcaneal QUS might not be a direct surrogate of femoral neck DXA BMD because it may measure the nature of the bone quality (including the bone strength and density) at the calcaneus, while central DXA is the standard measurement for diagnosing low bone density at the hip and spine. Also, variations in QUS reference ranges may exist across different devices, reference populations, and study populations.^31^ The reference ranges of QUS T-scores might be incomparable with a BMD classification by DXA T-scores, which could cause significant discrepancies in the classification of bone density;^32^ therefore, some investigators suggested a cutoff value of QUS T-scores of ≤−1.8 for osteoporosis^33^ versus ≤ −2.5 used in the study. The young reference populations for calculating T-scores between calcaneal QUS and femoral neck DXA were also not the same.

Significant loadings of the 9 screening tools onto 4 factors in both men and women indicated that these tools did not measure the presumed construct (i.e., fracture/osteoporosis risk). Different factors located by central DXA and calcaneal QUS measurements reflect that the 2 techniques may measure different bone natures and bone sites.^34^ The high loadings (≥0.9) of QUS parameters onto the same factor in both men and women suggested that these parameters consistently measure the same acoustic properties of the calcaneus. On the other hand, it was intriguing that lumbar spine and femoral neck DXA BMDs had only modest loading scores (0.64 and 0.65 in men and 0.58 and 0.60 in women, respectively) onto the same factor. This finding indicates that despite the 2 DXA measurements having measured a similar construct, some degree of discrepancy existed in the 2 DXA BMD measurements, compared to those simple screening tools with higher loadings (≥0.7) onto the underlying construct.

Several limitations of our study should be noted. First, the results cannot be generalized to all older people because a spectrum effect could have occurred. In addition to a healthy volunteer effect that may have existed in the study participants recruited from advertising posters and flyers, the exclusion of those patients with severe spondylosis and joint arthritis of the lower limbs may also have reduced the prevalence of osteoporosis in the study population and thus underestimated the overall clinimetric performances of the self-assessment tools. Moreover, despite the study results being separately presented for men and women, the spectrum effect might have differed between men and women because more female than male participants might not only reflect their health status but also their awareness of bone health. Second, cutoff values of the assessment tools, such as the ABONE, BWC, ORAI, OSIRIS, and SCORE, were based on Caucasian postmenopausal women only, and their appropriateness for older men in Taiwan or Asia needs to be further validated. Finally, the use of injurious falls as a surrogate for fractures needs to be further validated in future studies. Interrelationships among risks of falls, osteoporosis, and fractures are not clearly understood, and some environmental factors associated with falls but not related to fracture occurrences may have confounded the prediction validity.

## CONCLUSIONS

Simple self-assessment tools can serve as initial screening tools to rule out individuals who do not need further BMD testing or have no risk of a fracture; however, these simple tools may measure different constructs from central DXA, calcaneal QUS, and the FRAX. Further research is needed to investigate whether these tools do measure the risk of fracture/osteoporosis and have similar clinical performances between older men and women.
