# Comparisons of Different Screening Tools for Identifying Fracture/Osteoporosis Risk Among Community-Dwelling Older People: Erratum

**DOI:** 10.1097/01.md.0000494748.75305.53

**Published:** 2016-08-26

In the article “Comparisons of Different Screening Tools for Identifying Fracture/Osteoporosis Risk Among Community-Dwelling Older People”,^1^ which appeared in Volume 95, Issue 20 of *Medicine*, Table 1 was originally formatted incorrectly. The article has since been corrected online.

## References

[1] Chen SJ Chen YJ Cheng CH Comparisons of different screening tools for identifying fracture/osteoporosis risk among community-dwelling older people. *Medicine.* 2016 95:e3415. 27196447 10.1097/MD.0000000000003415 PMC4902389

